# Assessment of radionuclide levels, annual effective dose, and lifetime cancer risk from cultivated crop samples in Hainan Island, China

**DOI:** 10.3389/fpubh.2026.1863848

**Published:** 2026-06-26

**Authors:** Jun Chao, Zaiyun Zhu, Wenfang Meng, Xiao Luo, Chuanjian Wang, Jiao Li, Kai Liao

**Affiliations:** Hainan Provincial Center for Disease Control and Prevention (Hainan Academy of Preventive Medicine), Haikou, Hainan, China

**Keywords:** annual effective dose, crops, food safety, Hainan Island, lifetime cancer risk, natural radionuclides

## Abstract

**Objective:**

This study aims to evaluate the activity concentrations of natural radionuclides in major edible crops from Hainan Island, quantify the annual effective dose and lifetime cancer risk (ELCR) caused by dietary intake, and provide a scientific basis for local food safety regulation and radiation protection strategies.

**Methods:**

Sixteen representative crop samples (12 vegetables, 4 grains) were collected in 2025. High-purity germanium gamma spectrometry (HPGe) was used to measure the activity concentrations of ^228^Ra, ^226^Ra, and ^40^K. Combined with Chinese dietary consumption data and ICRP-recommended dose conversion coefficients, the annual effective doses for adults and children were calculated, and ELCR was estimated using a risk model.

**Results:**

Radionuclide activities followed the order ^40^K > ^228^Ra > ^226^Ra. The average ^40^K concentration was highest (vegetables: 121.517 (95% CI: 99.252–143.781) Bq/kg; grains: 146.375 (95% CI: −194.875–487.625) Bq/kg). ^226^Ra and ^228^Ra levels were below national regulatory limits. The mean annual effective doses from vegetable consumption were 0.116 (95% CI: 0.095–0.137) mSv/y for adults and 0.213 (95% CI: 0.169–0.257) mSv/y for children, grain consumption contributed 0.216 (95% CI: −0.304–0.736) mSv/y and 0.405 (95% CI: −0.593–1.403) mSv/y, respectively. Only one rice sample (G2) produced an annual effective dose for children (1.344 mSv/y) exceeding the 1 mSv/y reference level. ELCR was far below the ICRP recommended limit (2.5 × 10^−3^), with children’s risk approximately 1.8 times higher than that of adults.

**Conclusion:**

The radioactivity levels in Hainan crops are generally safe, and the radiation risk from dietary intake is within acceptable limits. Children require particular attention due to differences in metabolism and consumption rates. Long-term monitoring is recommended, especially for staple crops like rice, and further research should track the impact of agricultural practices on radionuclide migration.

## 1 Introduction

Public exposure to ionizing radiation primarily originates from natural radiation, with the radiation dose depending on geographical location and lifestyle (1). The main sources of natural radiation are natural radionuclides, including uranium-235, uranium-238, thorium-232 and their decay products, cosmogenic radionuclides like carbon-14 and tritium, and the isotope potassium-40, which constitute the majority of background radiation exposure (2, 3). Radionuclides released from nuclear weapons testing and nuclear power plant accidents can still be detected in the global environment, posing a significant radiation risk (4, 5). Potassium is an essential nutrient for the human body, while radium, thorium, and their decay products are not (6–8). These radionuclides can enter the human body through direct contact pathways such as skin penetration or wound entry, as well as through inhalation of radioactive gases or aerosols, and ingestion of contaminated food or water (9). Consuming radioactively contaminated food leads to internal exposure, the health effects of which depend primarily on the activity concentration of the ingested radionuclides, their retention time in the body, and the radiation sensitivity of the target organs (10–12). According to the latest assessment by the United Nations Scientific Committee on the Effects of Atomic Radiation (13), the global average annual effective dose from natural radiation sources is approximately 3 mSv, of which the dose contributed by ingesting radionuclides through food and drinking water is about 0.5 mSv (accounting for roughly 17%) (13).

With the public’s growing awareness of the health risks posed by both natural and artificial radiation, coupled with increasing demands for food safety, the issue of radionuclide contamination in food has become a focal point of research in recent years. Numerous studies have assessed natural radioactivity in food from different regions: in China, radionuclide levels have been measured in foods such as tea, rice, vegetables, and fish (14, 15); researchers in Vietnam estimated the activity concentrations of ^40^K, ^226^Ra, ^232^Th, and total *α* and total *β* activity in ten common cereal crops grown in Ho Chi Minh City, along with the annual effective dose resulting from the ingestion of these natural radionuclides (16); a study in South Korea analyzed ^40^K and ^137^Cs in rice and Chinese cabbage samples collected from 15 regions between 2014 and 2023, estimating annual effective doses of 7.53 μSv for rice and 10.1 μSv for Chinese cabbage, which correspond to approximately 0.7% of the natural background radiation (2,400 μSv/y) and about 5.8% of the total ingestion dose from food (290 μSv/y), respectively (17); in Türkiye, measurements of radionuclide concentrations in commonly consumed cereals, meat, dairy products, and bakery products showed the highest levels in cereals and bakery products (18); researchers used an HPGe gamma-ray spectrometer to determine the natural radioactivity levels in vegetable samples from Kirkuk City, Iraq, finding the highest concentrations of ^226^Ra and ^232^Th in root vegetables like garlic, and the highest 40 K content in the legume kidney beans, and subsequently evaluated the annual effective dose for adults, children (10 years old), and infants resulting from vegetable consumption (19).

Ingestion of natural radionuclides through the food chain represents a major source of internal exposure in humans. Accurate determination of their activity concentrations and estimation of the resulting effective dose are therefore crucial for safeguarding public health. Hainan Island, China’s second-largest island, is situated within the tropical zone between 18°10′N and 20°10′N. It borders the Beibu Gulf to the west and faces the South China Sea to the east and south. Characterized by a tropical monsoon climate, the island experiences long summers without winter and abundant sunshine and heat. With extended supply periods and high freshness, its winter melons and vegetables serve as a crucial “winter vegetable basket” for China. Although investigations into radionuclides in food have been conducted extensively both domestically and internationally, systematic research on major edible crops from Hainan Island remains scarce. This study, based on 2025 monitoring data for crops in Hainan Island, focuses on analyzing the distribution characteristics of the activity concentration of the natural radionuclide 40 K. Using dose conversion coefficients recommended by the International Commission on Radiological Protection (ICRP), we quantitatively calculated the annual effective dose for two key population groups: adults and children, and further quantified the associated lifetime cancer mortality risk. By comparing the assessment results with international safety standards and background radiation levels, we systematically evaluated the radiological safety of widely consumed crops in Hainan Island. The study aims to provide a scientific basis for local food safety regulation and the development of radiation protection strategies.

## 2 Experimental methods

### 2.1 Sample collection and treatment

**Figure 1:**
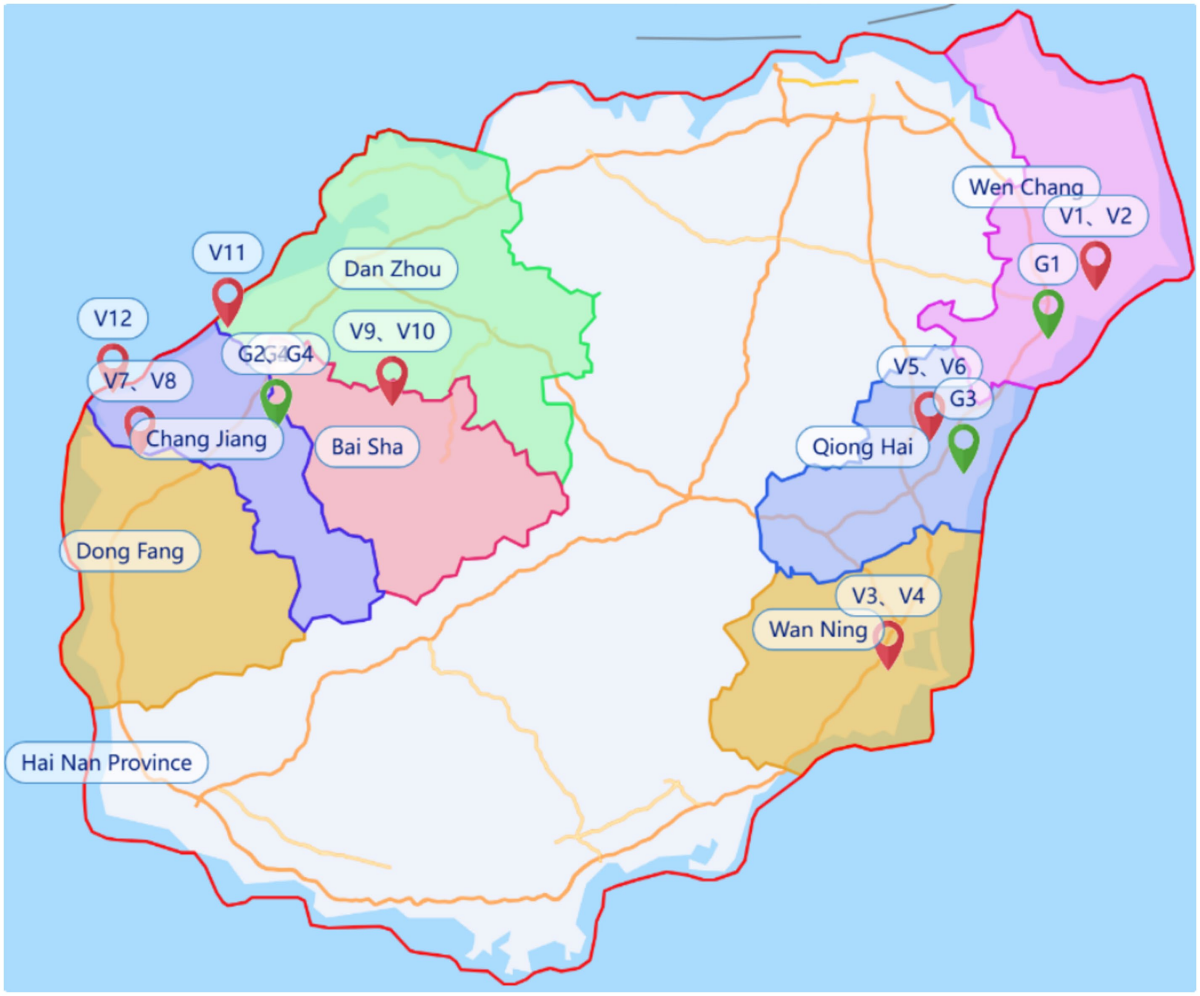
Schematic diagram of vegetable and grain collection sites.

Hainan Island has a permanent population of 10.55 million. Guided by local dietary habits, representative seasonal crops with high consumption rates were identified from seven counties (Wenchang, Wanning, Qionghai, Dongfang, Baisha, Danzhou, and Changjiang), and field samples were collected during their respective harvest seasons. A total of sixteen samples spanning two categories (vegetables and grains) were obtained. Specific characteristics of the collected samples (e.g., crop type, collection site, and food category) are summarized in Table 1, and the spatial distribution of sampling sites is shown in Figure 1.

**Table 1:** Sample collection information.

Sample Type	Location	Sample code	Longitude	Latitude	Sample name	Date
Vegetables	Wengchang	V1	110°46′56″E	19°38′8” N	Water spinach	2025.4.27
		V2	110°46′56″E	19°38′8” N	Pak choi	2025.4.27
	Wanning	V3	110°24′7″E	18°47′46”N	Water spinach	2025.7.9
		V4	110°24′7″E	18°47′46”N	Sweet potato leaves	2025.5.7
	Qionghai	V5	110°29′32″E	19°16′13”N	Water spinach	2025.6.11
		V6	110°29′32″E	19°16′13”N	Pak choi	2025.6.11
	Dongfang	V7	108°45′30″E	19°14′28”N	Mustard greens	2025.6.24
		V8	108°45′30″E	19°14′28”N	Shanghai bok choy	2025.6.24
	Baisha	V9	109°18′42″E	19°20′47”N	Water spinach	2025.6.4
		V10	109°18′42″E	19°20′47”N	Sweet potato leaves	2025.6.4
	Danzhou	V11	108°57′07″E	19°30′24”N	Mustard greens	2025.6.4
	Changjiang	V12	108°42′01″E	19°22′19”N	Water spinach	2025.5.19
Grain	Wenchang	G1	110°46′56″E	19°38′8”N	Rice​	2025.4.27
	Changjiang	G2	109°03′20″E	19°17′53”N	Rice​	2025.5.11
	Qionghai	G3	110°31′24″E	19°15′41”N	Sweet potato	2025.6.16
	Changjiang	G4	109°03′20″E	19°17′53”N	Sweet potato	2025.5.13

For each fresh sample, 10 kg were taken. Vegetable samples were cleaned of adhering soil and other debris, and were then sent to the laboratory along with grain samples for subsequent studies. In the laboratory, all vegetable samples were washed to remove adhering impurities, inedible parts, and soil particles. The samples were then air-dried, and the edible portions (leaves and stems) were separated. Following this, all samples (including grains) underwent the following processing: first, they were dried at room temperature and weighed, recording the fresh weight (mf); then, they were placed in clean ceramic trays and dried in an oven, with the temperature gradually increased to 105 °C, for 8 h until a constant weight was achieved, which was recorded as the dry weight (md) (20, 21). Both the fresh and dry weight values were used for calculating the specific activity concentration and effective dose of the crop samples.

All samples were ground into fine powder and passed through a 2 mm aperture steel laboratory sieve to achieve uniform particle size. Approximately 65 grams of each homogenized sample was then placed into a cylindrical Marinelli beaker (with a diameter of 75 mm and height of 70 mm), compacted, and sealed. The sample weight was calculated by the subtraction method for subsequent analysis. To ensure representativeness and result reproducibility, the same mass was used for each sample during measurement. The sealed samples were stored for at least 30 days prior to measurement, allowing the uranium and thorium series radionuclides to achieve secular equilibrium with their progeny (22, 23). This pretreatment step ensures the traceability of the activity concentration results and significantly reduces uncertainties that could arise from potential disequilibrium states (24).

### 2.2 Gamma spectrometric analysis

The activity concentrations of radionuclides in the samples were measured using a high-resolution gamma-ray spectrometer (Model GEM50P4, ORTEC). Each sealed sample was continuously measured for approximately 86,400 s, with triplicate measurements performed to obtain statistically reliable gamma-ray spectra. The gamma spectrometer is equipped with a coaxial p-type HPGe detector, which has a relative efficiency of 39.5% compared to a 3” × 3” NaI(Tl) crystal. The energy resolution (Full Width at Half Maximum) at 1332 keV (^60^Co) is 1.85 keV, and the integral background count rate for an 86,400-s measurement is 108 counts per minute (cpm) in the 50–2000 keV energy range. The high-purity germanium detector is shielded within a 10 cm thick lead chamber with an internal cavity measuring Φ29 cm × 74 cm to reduce the local radiation background.

Prior to measurements, the detection efficiency was calibrated using a multi-radionuclide standard source traceable to the National Institute of Metrology, China. The standard source contained ^241^Am, ^109^Cd, ^57^Co, ^139^Ce, ^131^I, ^133^Ba, ^51^Cr, ^134^Cs, ^137^Cs, ^54^Mn, ^88^Y, ^65^Zn, ^60^Co, ^22^Na, and ^40^K. The quality assurance and quality control (QA/QC) system encompasses both internal routine monitoring and external proficiency testing: on one hand, routine performance checks, background stability monitoring, and peak shape fitting verification are implemented for the HPGe gamma spectrometry system; on the other hand, regular participation in the ‘National Proficiency Testing Program for Gamma Spectrometric Analysis of Radionuclides’ organized by the National Institute for Radiological Protection, China CDC ensures the overall accuracy and reliability of the analytical data.

The activity of ^226^Ra in the samples was determined via the gamma-ray lines of ^214^Pb at energies of 295.2 keV and 351.9 keV, and the gamma-ray line of ^214^Bi at 609.31 keV. The activity concentration of ^228^Ra was determined by measuring the gamma-ray line of ^228^Ac at 911.2 keV. The activity concentration of ^40^K was obtained by measuring its gamma-ray line at 1460.8 keV. Uncertainties were determined according to the Guide to the Expression of Uncertainty in Nuclear Analytical Measurement (25).

During *γ*-ray spectrometry measurements, for cases where the background count (N_b_) is sufficiently large (i.e., >30), the minimum detectable activity (MDA) of a specific radionuclide in the sample can be calculated using Equation 1:

$$\mathrm{MDA}=\frac{k_{1–\alpha }+k_{1–\beta }}{\mathrm{\varepsilon Pm}}\sqrt{n_{b}[\frac{1}{T_{b}}+\frac{1}{T_{s}}]}$$ (1)

*k*_1 − *α*_ is the 1 − α percentile of the standard normal distribution (the significance level α is typically set to 5%, corresponding to *k*_1 − α_ = 1.645); *k*_1 − *β*_ is the 1 − *β* percentile of the standard normal distribution (the statistical power 1 – *β* is typically set to 95%, corresponding to *k*_1 − *β*_ = 1.645); *ε* is the full-energy peak detection efficiency for the *γ*-ray of interest, expressed in counts per second per becquerel [(counts/s)/Bq]; P is the emission branching ratio of the γ-ray of interest; m is the mass of the measured sample in kilograms (kg); n_b_ is the background count rate; *T*_b_ is the live time for background measurement in seconds (s); *T*_s_ is the live time for sample measurement in seconds (s).

### 2.3 Estimation of radiological hazard

#### 2.3.1 Ingestion effective dose

The annual effective ingestion dose (H) refers to the radiation dose absorbed or received by various organs of the body over 1 year due to the ingestion of natural radionuclides present in food. This parameter is used to describe the radiological risk associated with radiation exposure, balancing carcinogenic effects, life shortening, and genetic impacts. Differences in metabolic rates, body weight, and dietary habits influence the dose conversion and intake for different age groups. The annual effective ingestion dose accumulated by different age groups, specifically children (7–12 years old) and adults (>17 years old), due to the ingestion of radionuclides in food is estimated using the following formula 2 (26, 27):

$$H=\sum_{i=1}^{n}(A_{S}\times C_{\mathrm{Ann}})\times I_{\mathrm{DC}}$$ (2)

Where A_S_ is the specific activity concentration (Bq/kg) of the target radionuclide i, C_Ann_ is the annual average consumption (kg/y), and​ I_DC_ is the ingestion dose coefficient for the radionuclide (Sv/Bq). The annual average consumption (C_Ann_) values used in this study are based on the Chinese standard GBZ/T 200.4–2009 (28). The consumption rates for adults are: grains 164.25 kg/y and vegetables 131.4 kg/y. For children, the rates are: grains 98.55 kg/y and vegetables 94.9 kg/y. The values for the age group-specific ingestion dose conversion factors (*I*_DC_) are listed in Table 2 (29, 30). By estimating the age-related dose per unit intake, a more universally applicable dose assessment system is established. This approach helps refine the basis for dosimetric evaluation while enabling the extrapolation and supplementation of limited tissue concentration data (30).

**Table 2:** Dose conversion factors for ^226^Ra, ^228^Ra, and ^40^K used in determining annual effective dose for different age groups.

Age group	Dose conversion factors (mSv/Bq)		
	^226^Ra	^228^Ra	^40^K
Children (7–12 years)	8.0 × 10^−04^	3.9 × 10^−03^	1.3 × 10^−05^
Adults (>17 years)	2.8 × 10^−04^	6.9 × 10^−04^	6.2 × 10^−6^

#### 2.3.2 Excess lifetime cancer risk

Lifetime Cancer Risk (ELCR) refers to the probability of an individual developing cancer due to lifelong continuous exposure to low-dose ionizing radiation (31). The ELCR value induced by the ingestion of radionuclides (^226^Ra, ^228^Ra, and ^40^K) in food is calculated from the annual effective dose using the following formula 3 (32):

ELCR=H×L×RF (3)

where H is the annual effective ingestion dose (Sv/y), L is the average life expectancy (70 years), and RF is the risk coefficient for stochastic cancer effects per unit dose. The risk factor for stochastic effects at low doses is defined by the International Commission on Radiological Protection (ICRP) as 0.05 Sv^−1^ (32). To account for variations in consumption behavior and dose conversion factors with age, the annual ingestion dose for each radionuclide was estimated separately for children and adults, and the Excess Lifetime Cancer Risk (ELCR) was subsequently assessed based on these estimates.

## 3 Results and discussion

### 3.1 Activity concentrations of natural radionuclides in crops

The minimum detectable activity (MDA) and radionuclide concentrations of ^226^Ra, ^228^Ra, and ^40^K were determined in 16 crop samples (12 vegetables and 4 grains) collected from Hainan Island (Tables 3, 4). In the calculation of activity concentrations, values below the MDA were substituted with MDA/2.

**Table 3:** Minimum detectable activity (MDA) for ^228^Ra, ^226^Ra, and ^40^K activity concentrations in vegetable and grain samples from Hainan Island.

Sample type	Sample code	Sample name	^228^Ra	^226^Ra	^40^K
Vegetables	V1	Water spinach	0.074	0.037	1.700
	V2	Pak choi	0.092	0.053	3.000
	V3	Water spinach	0.053	0.031	1.700
	V4	Sweet potato leaves	0.061	0.019	1.100
	V5	Water spinach	0.045	0.014	1.200
	V6	Pak choi	0.055	0.029	1.800
	V7	Mustard greens	0.066	0.033	1.800
	V8	Shanghai bok choy	0.035	0.012	0.800
	V9	Water spinach	0.072	0.033	2.000
	V10	Sweet potato leaves	0.076	0.043	2.100
	V11	Mustard greens	0.044	0.013	1.000
	V12	Water spinach	0.067	0.038	2.000
Grain	G1	Rice	0.146	0.060	2.400
	G2	Rice	0.500	0.26	11.000
	G3	Sweet Potato	0.086	0.033	1.600
	G4	Sweet Potato	0.094	0.070	1.400

**Table 4:** Activity concentrations of ^228^Ra,^226^Ra and ^40^K in vegetables and grains samples from Hainan Island (Bq/kg).

Sample Type	Sample code	Sample name	^228^Ra	^226^Ra	^40^K
Vegetables	V1	Water spinach	0.249	0.181	134.800
	V2	Pak choi	0.263	0.168	194.000
	V3	Water spinach	0.026*	0.016*	116.600
	V4	Sweet potato leaves	0.394	0.104	83.500
	V5	Water spinach	0.055	0.023	108.600
	V6	Pak choi	0.028*	0.014*	129.900
	V7	Mustard greens	0.196	0.016*	127.400
	V8	Shanghai bok choy	0.071	0.029	68.500
	V9	Water spinach	0.254	0.101	141.000
	V10	Sweet potato leaves	0.139	0.044	120.600
	V11	Mustard greens	0.122	0.020	77.300
	V12	Water spinach	0.087	0.088	156.000
	Minimum		<MDA	<MDA	68.500
	Maximum		0.394	0.181	194.0
		Mean ± 95% CI	0.157 ± 0.073	0.067 ± 0.038	121.517 ± 22.265
Grain	G1	Rice	0.073*	0.030*	40.500
	G2	Rice	1.860	0.394	467.000
	G3	Sweet potato	0.181	0.046	60.300
	G4	Sweet potato	0.047*	0.035*	17.700
	Minimum		<MDA	<MDA	17.700
	Maximum		1.860	0.394	467.000
	Mean ± 95% CI		0.540 ± 1.403	0.126 ± 0.284	146.375 ± 341.245

^*^Activity concentration below the minimum detectable activity; reported value = MDA/2, used for calculation purposes only.

The analysis indicated that among the vegetable samples, ^228^Ra was not detected (below the detection limit) in 2 samples (sample codes V3, V6). For the 10 samples where ^228^Ra was detected, the lowest concentration was found in a water spinach sample (0.055 Bq/kg, sample code V5), while the highest concentration was observed in another water spinach sample (0.394 Bq/kg, sample code V4), with a mean value of 0.157 (95% CI: 0.084–0.230) Bq/kg.

^226^Ra was detected in 9 vegetable samples, with 3 samples showing activity below the detection limit (sample codes V3, V6, V7). The lowest concentration was found in a mustard sample (0.020 Bq/kg, sample code V11), and the highest in a water spinach sample (0.181 Bq/kg, sample code V1), with a mean value of 0.067 (95% CI: 0.029–0.105) Bq/kg.

For ^40^K in the 12 vegetable samples, the lowest concentration was observed in a pak choi sample (68.500 Bq/kg, sample code V8), and the highest in a green leafy vegetable sample (194.000 Bq/kg, sample code V2), with a mean value of 121.517 (95% CI: 99.252–143.781) Bq/kg.

In grain samples, ^228^Ra was detected in 2 samples, with the lowest concentration found in sweet potato (0.181 Bq/kg, sample code G3) and the highest in paddy rice (1.860 Bq/kg, sample code G2), yielding a mean value of 0.540 (95% CI: −0.863–1.943) Bq/kg. The remaining 2 samples showed activity below the detection limit (sample codes G1, G4).

^226^Ra was detected in 2 grain samples, while the other 2 were below the detection limit (sample codes G1, G4). The lowest concentration was observed in sweet potato (0.046 Bq/kg, sample code G3) and the highest in paddy rice (0.394 Bq/kg, sample code G2), with a mean value of 0.126 (95% CI: −0.158–0.410) Bq/kg.

For ^40^K, the lowest concentration was found in a sweet potato sample (17.700 Bq/kg, sample code G4) and the highest in a paddy rice sample (467.000 Bq/kg, sample code G2), resulting in a mean value of 146.375 (95% CI: −194.875–487.625) Bq/kg.

The results of this study indicate that the activity concentrations of detected natural radionuclides in all samples vary depending on the radionuclide species, place of origin, and sample type, exhibiting an overall distribution pattern of ^40^K > ^228^Ra > ^226^Ra. This is primarily attributed to the relatively high natural abundance of potassium in the Earth’s crust and its role as an essential nutrient for plant growth, which facilitates its bioaccumulation in plants through metabolic processes (33). In some crop samples, the activity concentrations of ^228^Ra and ^226^Ra were below the Minimum Detectable Activity (MDA). However, this does not necessarily indicate the absolute absence of these radionuclides. It is noted that weak characteristic full-energy peaks may be obscured and thus not effectively identifiable due to limitations posed by instrument background and system MDA (34). Furthermore, variations in the activity concentrations of natural radionuclides exist even within the same type of crop. These variations are primarily associated with factors such as the soil matrix in the growing environment, irrigation water quality, atmospheric deposition, and the application of agricultural fertilizers (especially potassic fertilizers).

Although ^40^K exhibited the highest activity concentration among the radionuclides in these food samples, no specific guidance limits have been established for ^40^K concentration in food. This is because potassium is an essential macronutrient for the human body. As a natural isotope of potassium, the content of ^40^K in the body remains in a dynamic equilibrium regulated by potassium metabolism and the stable concentration distribution of potassium within the body. It is effectively maintained at a constant level through homeostatic mechanisms (35, 36).

The activity concentrations of the radionuclides ^228^Ra, ^226^Ra, and ^40^K in crops from this study were compared with those from similar existing research in China and other countries, as summarized in Table 5. The activity concentrations of ^228^Ra, ^226^Ra, and ^40^K in vegetable samples were generally comparable to or slightly lower than the natural background values in China and those reported in Singapore and Croatia (37–39), but were significantly lower than those reported in Brazil and Ho Chi Minh City, Vietnam (16, 40). The maximum activity concentrations of the three radionuclides in grain samples were notably higher than the background values in China (37). Compared to values from Serbia, Thailand, and Pakistan, the ^226^Ra values in those studies were an order of magnitude higher than those in the present study. The 40 K values, except for the significantly higher level in Serbia, were generally comparable to those found in this study in Thailand and Pakistan (3, 37, 41, 42).

**Table 5:** Activity concentration ranges of ^226^Ra, ^228^Ra and ^40^K in vegetables and grains from the literature (Bq/kg).

Location	Sample Type	^228^Ra	^226^Ra	^40^K	References
China	Vegetables	<MDA-0.394	<MDA-0.181	68.5–194.0	This study
China		ND–0.466	0.0392–0.122	–	(36)
China GB 14882–1994		–	11	–	(42)
Vietnam		–	0.04–2.58	74–1867	(16)
Singapore		0.036–0.419	0.029–0.155	108–230	(37)
Croatia		0.130–0.409	0.060–0.328	55–193	(38)
Brazil		<0.26–36.27	<0.14–3.77	48.64–165.79	(39)
China	Grain	<MDA-1.860	<MDA-0.394	17.7–467.0	This study
China		ND–0.133	0.0116–0.0207	–	(36)
China GB 14882–1994		–	14	–	(42)
Serbia		–	3.25–8.66	428.82–1424.79	(3)
Thailand		–	1.48–4.81	6.85–63.5	(40)
Pakistan		–	1.67 ± 1.19	88.51 ± 11.65	(41)

The observed variations in radioactivity levels among crops cultivated in different countries, including China, can be attributed to differences in climate, soil, cultivation practices, anthropogenic activities near the farmlands, and the specific types of crops evaluated in the studies. According to the current national standards for limits of radionuclides in foods in China, the maximum permitted levels of ^226^Ra are 14 Bq/kg for grains and 11 Bq/kg for vegetables, respectively. The activity concentrations of this radionuclide in all crop samples analyzed in this study were below these regulatory limits (43).

### 3.2 Estimation of annual effective dose and lifetime Cancer risk

Two parameters, the annual effective ingestion dose and the lifetime cancer risk, were used to assess the radiological health risk to Hainan Island residents from consuming crops. These parameters reflect both the annual radiation burden to consumers and the potential long-term stochastic health effects. In the calculation of activity concentrations, values below the MDA were substituted with MDA/2. The annual effective dose results for adults and children, aggregated by crop category, are presented in Table 6.

**Table 6:** Assessment results of the effective ingestion dose for adults and children from the studied crop (vegetables and grains) samples.

Sample Type	Sample code	Annual effective intake for adults (mSv/y)				Annual effective intake for children (mSv/y)			
		^228^Ra	^226^Ra	^40^K	Sum	^228^Ra	^226^Ra	^40^K	Sum
Vegetables	V1	0.022	0.007	0.110	0.139	0.092	0.014	0.166	0.272
	V2	0.024	0.006	0.158	0.188	0.097	0.013	0.239	0.349
	V3	0.002	0.001	0.095	0.098	0.010	0.001	0.144	0.155
	V4	0.036	0.004	0.068	0.108	0.146	0.008	0.103	0.257
	V5	0.005	0.001	0.088	0.094	0.020	0.002	0.134	0.156
	V6	0.002	0.001	0.106	0.109	0.010	0.001	0.160	0.171
	V7	0.018	0.001	0.104	0.123	0.072	0.001	0.157	0.230
	V8	0.006	0.001	0.056	0.063	0.026	0.002	0.084	0.112
	V9	0.023	0.004	0.115	0.142	0.094	0.008	0.174	0.276
	V10	0.013	0.002	0.098	0.113	0.051	0.003	0.149	0.203
	V11	0.011	0.001	0.063	0.075	0.045	0.002	0.095	0.142
	V12	0.008	0.003	0.127	0.138	0.032	0.007	0.192	0.231
	Mea*n* ± 95% CI	0.014 ± 0.007	0.003 ± 0.001	0.099 ± 0.018	0.116 ± 0.021	0.058 ± 0.027	0.005 ± 0.003	0.150 ± 0.028	0.213 ± 0.044
Grain	G1	0.008	0.001	0.041	0.050	0.028	0.002	0.052	0.082
	G2	0.211	0.018	0.476	0.705	0.715	0.031	0.598	1.344
	G3	0.021	0.002	0.061	0.084	0.070	0.004	0.077	0.151
	G4	0.005	0.002	0.018	0.025	0.018	0.003	0.023	0.044
	Mea*n* ± 95% CI	0.061 ± 0.159	0.006 ± 0.013	0.149 ± 0.348	0.216 ± 0.520	0.208 ± 0.539	0.010 ± 0.022	0.188 ± 0.437	0.405 ± 0.998

**Figure 2:**
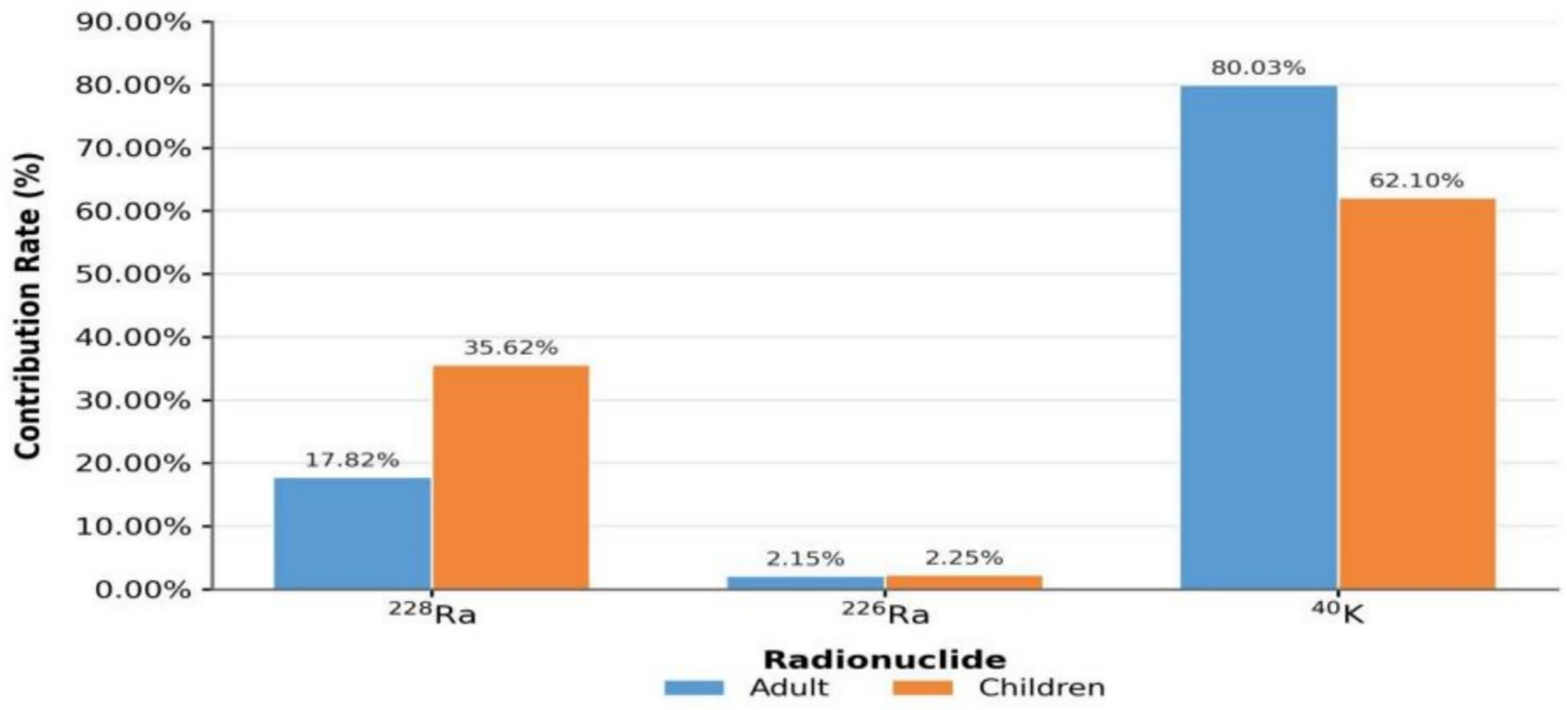
Contribution rates of radionuclides (^228^Ra, ^226^Ra, ^40^K) to total radiation exposure for adults and children.

The contribution of natural radionuclides to the total annual effective dose is illustrated in Figure 2. Among the total annual effective dose attributed to crops, ^40^K made the highest contribution. However, potassium levels in the human body are tightly regulated through metabolism to maintain a stable state; therefore, the intake of ^40^K does not typically pose a health risk (44). Notably, the contribution of ^228^Ra and ^226^Ra to radiation exposure was significantly higher for children than for adults. This is a critical difference that must be considered in health risk assessments.

The mean annual effective dose resulting from the consumption of crops (vegetables and grains) was approximately 332 μSv/y for adults and 618 μSv/year for children. Grains contributed significantly more than vegetables, a difference stemming from their higher average activity concentrations and assumed intake rates. The estimated annual effective doses for adults (approximately 332 μSv/y) and children (approximately 618 μSv/year) align with the commonly reported range of 300–700 μSv/y for mixed diets. Grains made the largest contribution to the ingestion dose, primarily due to the assumed higher per capita annual consumption of grains compared to vegetables.

With the exception of the pediatric group sample G2, which exhibited an annual effective intake dose of 1.344 mSv/y—slightly exceeding the international reference level of 1 mSv/y—all other crop samples remained within the prescribed limits. This indicates that the overall dietary exposure levels estimated in this study are consistent with global patterns. Analysis of the monitoring results reveals that the elevated dose in sample G2 is primarily attributed to an anomalously high activity concentration of ^40^K. Given that ^40^K is an essential natural radionuclide for plant growth, its enrichment is likely closely related to the historical application of potassium fertilizer for this specific crop batch. It is noteworthy that such anomalies were observed only in a single sample, representing a localized case that does not compromise the assessment of the overall regional safety level. It should also be noted that the findings of this study are based on the analysis of collected samples rather than the actual consumed state. In reality, culinary preparation processes such as washing, boiling, and baking can reduce radionuclide concentrations. Consequently, the actual annual effective ingestion dose to the public is likely lower than the values estimated in this assessment.

Table 7 presents the annual effective dose and lifetime cancer risk for different age groups from vegetable samples. Accordingly, for adults, the annual effective dose ranged from 0.063 to 0.188 mSv/y, while the lifetime cancer risk ranged from 0.222 × 10^−3^ to 0.658 × 10^−3^. The mean annual effective dose and mean lifetime cancer risk for adults were 0.116 (95% CI: 0.095–0.137) mSv/y and 0.405 (95% CI: 0.331–0.279) × 10^−3^, respectively. For children, the annual effective dose ranged from 0.112 to 0.349 mSv/y, and the lifetime cancer risk ranged from 0.395 × 10^−3^ to 1.223 × 10^−3^. The mean annual effective dose and mean lifetime cancer risk for children were 0.213 (95% CI: 0.169–0.257) mSv/y and 0.746 (95% CI: 0.593–0.899) × 10^−3^, respectively.

**Table 7:** Annual effective dose and lifetime cancer risk for different age groups (adults and children) from vegetable samples collected in Hainan Island.

Sample code	Annual effective dose (mSv/y)		Lifetime cancer risk×10^−3^	
	Adult	Children	Adult	Children
V1	0.139	0.272	0.487	0.953
V2	0.188	0.349	0.658	1.223
V3	0.098	0.155	0.332	0.503
V4	0.108	0.257	0.376	0.898
V5	0.094	0.156	0.330	0.546
V6	0.109	0.171	0.370	0.561
V7	0.123	0.230	0.426	0.804
V8	0.063	0.112	0.222	0.395
V9	0.142	0.276	0.496	0.965
V10	0.113	0.203	0.394	0.712
V11	0.075	0.142	0.262	0.497
V12	0.138	0.231	0.484	0.810
Mean ± 95% CI	0.116 ± 0.021	0.213 ± 0.044	0.405 ± 0.074	0.746 ± 0.153

Table 8 presents the annual effective dose and lifetime cancer risk for different age groups from grain samples. Accordingly, for adults, the annual effective dose ranged from 0.025 to 0.705 mSv/y, while the lifetime cancer risk ranged from 0.088 × 10^−3^ to 2.468 × 10^−3^. The mean annual effective dose and mean lifetime cancer risk for adults were 0.216 (95% CI: −0.304–0.736) mSv/y and 0.756 (95% CI: −0.590–2.102) × 10^−3^, respectively. For children, the annual effective dose ranged from 0.044 to 1.344 mSv/y, and the lifetime cancer risk ranged from 0.154 × 10^−3^to 4.705 × 10^−3^. The mean annual effective dose and mean lifetime cancer risk for children were 0.405 (95% CI: −0.593–1.403) mSv/y and 1.392 (95% CI: −1.214–3.998) × 10^−3^, respectively.

**Table 8:** Annual effective dose and lifetime cancer risk for different age groups (adults and children) from grain samples collected in Hainan Island.

Sample code	Annual effective dose (mSv/y)		Lifetime cancer risk×10^−3^	
	Adult	Children	Adult	Children
G1	0.050	0.082	0.175	0.182
G2	0.705	1.344	2.468	4.705
G3	0.084	0.151	0.294	0.527
G4	0.025	0.044	0.088	0.154
Mean ± 95% CI	0.216 ± 0.520	0.405 ± 0.998	0.756 ± 1.346	1.392 ± 2.606

**Figure 3:**
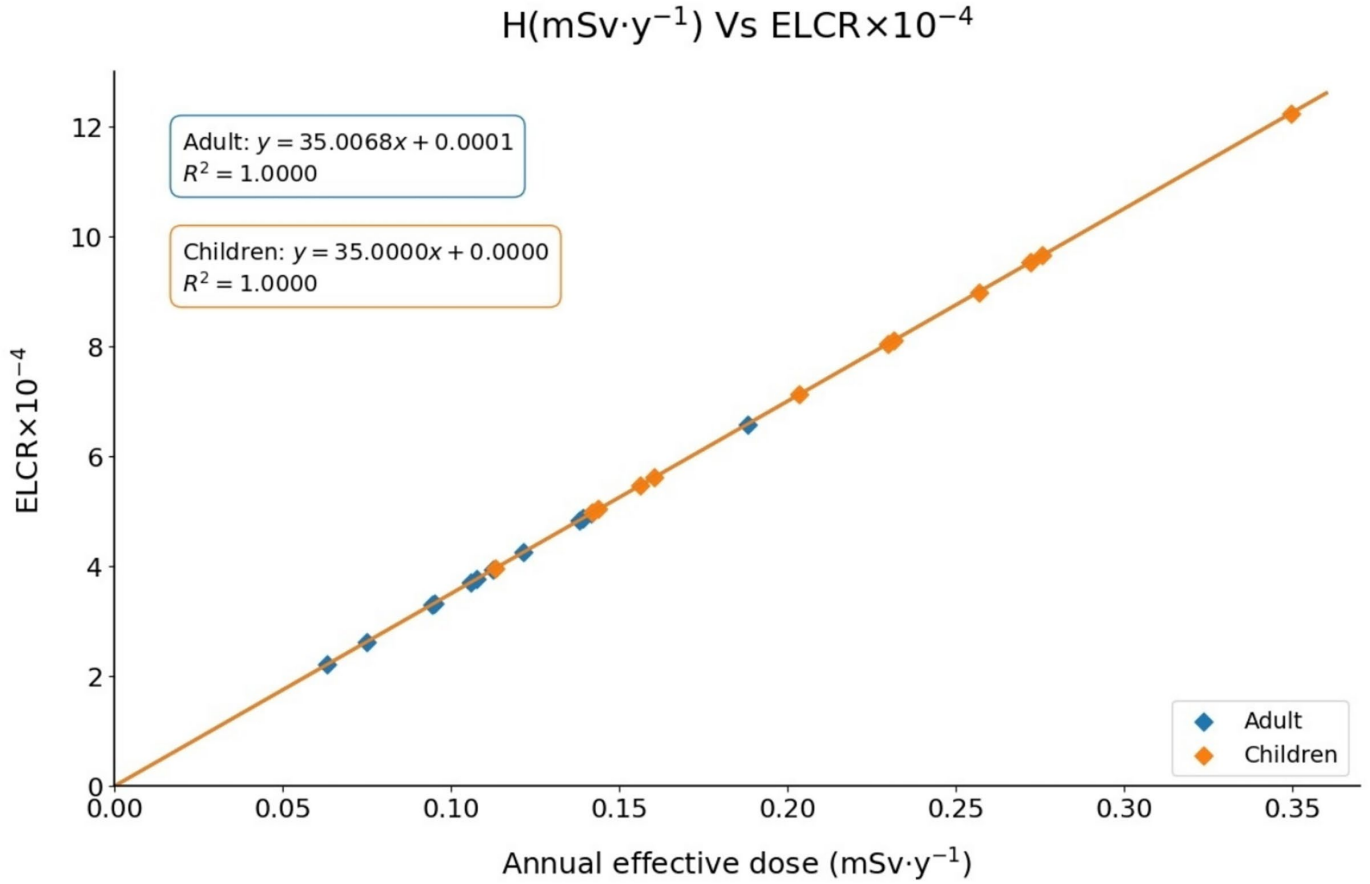
Correlation between annual effective dose (H) and excess lifetime cancer risk (ELCR).

From the analysis of annual effective dose and lifetime cancer risk for adults and children consuming the agricultural product samples, as presented in Tables 7, 8, and Figure 3, it was found that children exhibited the highest annual effective dose and lifetime cancer risk. Furthermore, the annual effective dose from grain samples was slightly higher than that from vegetable samples, which is primarily attributed to the assumed higher per capita annual consumption of grains compared to vegetables.

A comparison of the estimated lifetime cancer risk (ELCR) associated with crop consumption in this study with values reported in the relevant literature shows that the ELCR for vegetables was lower than the results from studies in Singapore and Bangladesh (38, 45). The ELCR for grain samples was comparable to findings from studies in Ghana, Malaysia, and Iraq (46–48). However, this risk is considered low for the general adult population. Due to differences in metabolic rates and dietary intake, children face a higher cancer risk. Figure 3 illustrates the relationship between the annual effective dose (H) and the excess lifetime cancer risk (ELCR) for both adult and child groups, showing a strong positive linear correlation. This indicates that an increase in annual effective dose is proportionally associated with an increase in radiation risk. The figure also demonstrates that both the annual effective dose and the corresponding ELCR levels are generally higher for the child group than for the adult group.

Furthermore, compared to the standard value for cancer risk (2.5 × 10^−3^) established by the International Commission on Radiological Protection (49), the mean lifetime cancer risk levels estimated in this study are substantially lower. Therefore, the consumption of the grain and vegetable samples investigated in this study does not pose a radiological health risk to the public.

## 4 Conclusion

In this study, we evaluated the activity concentrations of natural radionuclides in crops (vegetables and grains) cultivated in Hainan Island using gamma spectroscopy. The natural radionuclides ^228^Ra, ^226^Ra, and ^40^K were detected in a total of 12 vegetable and 4 grain samples collected. Although significant differences in activity concentrations were observed among different food categories, the levels of ^228^Ra and ^226^Ra were generally low, while ^40^K showed relatively higher concentrations, which showed relatively higher concentrations in the crops. Compared with findings from similar studies in domestic and international literature, the activity concentrations reported in this study were generally lower or comparable.

Both the estimated annual effective doses and the excess lifetime cancer risks (ELCR) for children and adults were below the reference levels recommended by the United Nations Scientific Committee on the Effects of Atomic Radiation (UNSCEAR) and the International Commission on Radiological Protection (ICRP). With the exception of one rice sample that exceeded the reference level for children, the overall radiological risk from the sampled crops appears low. However, the presence of this outlier warrants further targeted investigation of rice cultivated in the affected areas. Furthermore, the relatively higher annual effective ingestion dose for the child group highlights the importance of considering age as a key factor in dietary radiation exposure assessment. It is recommended to establish a long-term monitoring mechanism, with particular attention to staple crops with high consumption rates, such as rice, and to track the impact of agricultural practices (e.g., fertilizer use) on radionuclide transfer.

In summary, the radiological health risk to local residents from ingesting radionuclides through the consumption of locally grown crops is minimal. These findings can serve as a reference baseline for future assessments of radiological hazards in agricultural products, thereby contributing to ensuring food safety in Hainan Island.

Nevertheless, certain limitations should be acknowledged. Due to the uneven sampling strategy and the relative scarcity of grain samples, the representativeness of the dataset is somewhat constrained. This may affect the robustness and generalizability of the risk assessment results, particularly regarding the risk estimates for grain ingestion pathways. Future studies are needed to expand the sample size and optimize the spatial representativeness of the sampling layout to validate and refine these conclusions.

## Data Availability

The original contributions presented in the study are included in the article/supplementary material, further inquiries can be directed to the corresponding author.
